# Antibiotikaverschreibung in urologischen Kliniken Deutschlands: Ergebnisse einer Querschnittstudie

**DOI:** 10.1007/s00120-024-02469-2

**Published:** 2024-11-19

**Authors:** Winfried V. Kern, Jürgen Baumann, Gesche Först, Evelyn Kramme, Michaela Steib-Bauert, Jennifer Kranz, Giuseppe Magistro, Katja de With

**Affiliations:** 1 Klinik für Innere Medizin II, Abteilung Infektiologie, Universitätsklinikum Freiburg, Freiburg, Deutschland; 2 Medizinische Fakultät, Universität Freiburg, Freiburg, Deutschland; 3 Akademie für Infektionsmedizin e. V., Berlin, Deutschland; 4 Apotheke, medius Kliniken, Ostfildern, Deutschland; 5 Institut für Pharmazeutische Wissenschaften, Universität Freiburg, Freiburg, Deutschland; 6 Klinik für Infektiologie, Universitätsklinikum Schleswig-Holstein Campus Lübeck, Lübeck, Deutschland; 7 Klinik für Urologie und Kinderurologie, Uniklinik RWTH Aachen, Aachen, Deutschland; 8 Klinik und Poliklinik für Urologie, Universitätsklinikum Halle (Saale), Halle (Saale), Deutschland; 9 Klinik für Urologie, Asklepios Westklinikum GmbH, Hamburg, Deutschland; 10 Institut für Klinische Infektiologie, Universitätsklinikum Carl Gustav Carus an der TU Dresden, Dresden, Deutschland; 11 Abteilung Infektiologie, Universitätsklinikum Freiburg, Hugstetter Straße 55, 79106 Freiburg, Deutschland

**Keywords:** Pharmakoepidemiologie, Antibiotikaanwendung im Krankenhaus, Fluorchinolone, Cotrimoxazol, Aminoglykoside, Pharmacoepidemiology, Hospital antibiotic use, Fluoroquinolones, Cotrimoxazole, Aminoglycosides

## Abstract

**Hintergrund:**

Die Antibiotikaverordnung bei stationären Patienten unterscheidet sich je nach Fachrichtung sowohl hinsichtlich der Intensität als auch bezüglich des Wirkstoffspektrums.

**Ziel der Arbeit:**

Es wird eine Analyse und Bewertung der aktuellen Daten zur Antibiotikaverbrauchsdichte (AD) in deutschen urologischen Fachabteilungen durchgeführt.

**Material und Methoden:**

Die Antibiotikaverordnungsdaten von 107 urologischen Fachabteilungen wurden für den Zeitraum 2022/23 ausgewertet. Die Berechnung der AD erfolgte als Tagesdosen (festgelegt nach Empfehlungen für erwachsene stationäre Patienten, sog. „recommended daily doses“, RDD) pro 100 Pflegetage (RDD/100).

**Ergebnisse:**

Die AD betrug im Median 71 RDD/100 mit einer großen Spannweite von minimal 15,9 bis maximal 138,7 RDD/100. Es gab keine signifikanten Unterschiede nach Krankenhausgröße. Fluorchinolone waren mit einer medianen AD von 6,0 RDD/100 nach Cephalosporinen der dritten bzw. vierten Generation (Median 16,2 RDD/100), Aminopenicillin/Betalaktamaseinhibitor-Kombinationen (Median 10,8 RDD/100) und Piperacillin-Tazobactam bzw. Piperacillin (Median 8,9 RDD/100) die viertstärkste Substanzgruppe. Das Verhältnis Penicilline zu Cephalosporinen schwankte zwischen 6:94 und 98:2 (insgesamt 52:48). Aminoglykoside (< 1 %) und intravenöses Fosfomycin (< 0,1 %) hatten nur einen sehr kleinen Anteil an den Verordnungen. Cotrimoxazol (Median 4,0 RDD/100) wurde seltener als Fluorchinolone verordnet. Der Anteil oraler Antibiotika betrug insgesamt 44,7 % und schwankte nach Krankenhausgröße wenig. Dabei wurden Fosfomycin, Pivmecillinam, Nitrofurantoin und Nitroxolin deutlich seltener verordnet als orale Betalaktame, Fluorchinolone und Cotrimoxazol.

**Schlussfolgerung:**

Die AD in der Urologie schwankte auch 2022/23 erheblich. Betalaktame wurden mit Abstand am häufigsten verordnet. Fluorchinolone – vielfach oral – werden weiterhin eingesetzt, ihr Verbrauch schwankt ähnlich der gesamten AD erheblich und unabhängig von der Krankenhausgröße. Die bei der akuten unkomplizierten Zystitis empfohlenen Antibiotika spielen mengenmäßig im stationären Setting eine untergeordnete Rolle. Penicilline und Cotrimoxazol sollten vermehrt als Behandlungsalternative berücksichtigt werden. Auch intravenöses Fosfomycin oder Aminoglykoside sollten als Optionen bei ansonsten resistenten Erregern betrachtet werden.

## Abstract (en)

**Background:**

The patterns and intensity of inpatient antibiotic prescribing vary according to medical specialty.

**Objectives:**

Analysis of recent data on antibiotic use density in hospital departments of urology in Germany.

**Methods:**

Annual surveillance data of 107 departments for the period 2022/2023 were evaluated. We used a daily dose definition adapted for adult hospitalized patients (recommended daily doses, RDD), and 100 patient days as the denominator (RDD/100).

**Results:**

The overall median antibiotic use density was 71 RDD/100 with a wide range between 15.9 and 138.7 RDD/100 but no significant differences according to hospital size. Fluoroquinolones (median 6.0 RDD/100) were prescribed as the fourth most frequent antibiotic class after broad-spectrum cephalosporins (median 16.2 RDD/100), aminopenicillin/beta-lactamase inhibitor combinations (median 10.8 RDD/100), and broad-spectrum penicillins (piperacillin-tazobactam and piperacillin) (median 8.9 RDD/100). The ratio between penicillin and cephalosporin RDD per hospital ranged from 6:94 to 98:2 (overall 52:48). The proportion of aminoglycosides (< 1%) and parenteral fosfomycin (< 0.1%) was very small. Cotrimoxazole (median 4.0 RDD/100) was less frequently prescribed than fluoroquinolones. The proportion of oral agents was 44.7% overall, with only small differences according to hospital size. Oral fosfomycin, pivmecillinam, nitrofurantoin, and nitroxoline were much less frequently prescribed than oral beta-lactams, fluoroquinolones, and cotrimoxazole.

**Conclusion:**

The overall antibiotic use density in urological hospital departments varied substantially in 2022/2023. Beta-lactam antibiotics were the most frequently used antibiotics, while fluoroquinolones (often as oral agents) continued to be prescribed with a large range similar to overall antibiotic use and independent of hospital size. Inpatient prescribing of the agents recommended and typically used for uncomplicated cystitis was rare. Penicillins and cotrimoxazole should more often be considered as the treatment option. Aminoglycosides and parenteral fosfomycin should be discussed in cases of otherwise drug-resistant pathogens.

## Einleitung

Seit mehreren Jahren wird in vielen Ländern eine Erfassung und Bewertung des Antibiotikaverbrauchs im ambulanten und stationären Setting empfohlen. Für Krankenhäuser in Deutschland ist dies mit der Änderung des Infektionsschutzgesetzes im Jahre 2011 eine Verpflichtung geworden [1, 2] und auch per S3-Leitlinie der Arbeitsgemeinschaft der Wissenschaftlichen Medizinischen Fachgesellschaften (AWMF) empfohlen [3]. Demnach sollen Art und Umfang des Antibiotikaverbrauchs in aggregierter Form fortlaufend erfasst, aufgezeichnet und bewertet werden. Hintergrund ist ein seitens Wissenschaft, Ärzteschaft und zuletzt auch Politik gefordertes Monitoring im Zusammenhang mit der zunehmenden Entwicklung von Antibiotikaresistenzen weltweit. Eine Erfassung von landesweiten Verbrauchsmengen in Tagesdosen pro Kopf ist dabei für die ambulante Medizin vielerorts zur Routine geworden und wird für Länder der Europäischen Union (EU) regelmäßig veröffentlicht [4]. Für den Krankenhausbereich haben sich eine zuverlässige Erfassung und vergleichende Darstellung des Antibiotikaverbrauchs mit der Möglichkeit eines Benchmarkings bisher schwierig gestaltet. In Deutschland sind hierzu zwei nicht-kommerzielle Surveillance-Systeme verfügbar, einmal das sog. „AVS“ (Antibiotika-Verbrauchssurveillance) des Robert Koch Instituts in Kooperation mit der Charité [5], zum anderen die Antiinfektiva-Surveillance des Bundesverbands Deutscher Krankenhausapotheker in Kooperation mit der Deutschen Gesellschaft für Infektiologie und dem Universitätsklinikum Freiburg („ADKA-if-DGI-Surveillance“; [1, 6, 7]). Die bisherigen Berichte und Auswertungen des letztgenannten Systems zeigen, dass in den urologischen Fachbereichen die Antibiotikaverbrauchsdichte (AD) vergleichsweise hoch gewesen ist (www.antiinfektiva-surveillance.de). In den Analysen anderer Länder (z. B. Schweiz, Schweden, Dänemark, Niederlande u. a.) ist diese Auffälligkeit bisher nicht beschrieben [8–11]. Oft beinhalten die entsprechenden Berichte lediglich eine Unterscheidung zwischen Normal- und Intensivstation und regionale Betrachtung, nicht jedoch eine weitergehende Betrachtung auch der Fachdisziplinen, die für lokale Empfehlungen und Antibiotic-stewardship-Ziele und -Maßnahmen von Bedeutung ist.

Wir haben daher aktuelle Antibiotikaverbrauchsdaten speziell für urologische Fachabteilungen separat ausgewertet. Eine wichtige Fragestellung war, wie sich die aktuelle Spannweite der Verbrauchsdichte von Fluorchinolonen und alternativen Substanzen zur Behandlung von Harnwegsinfektionen im Vergleich der urologischen Fachabteilungen darstellt. Harnwegsinfektionen gehören nach wie vor zu den häufigsten Infektionen weltweit [12, 13]. Aktuelle Therapieempfehlungen sind verfügbar ([14, 15]; s. auch: https://register.awmf.org/de/leitlinien/detail/043-044%20KF). Eine sehr heterogene Verschreibungspraxis in deutschen Kliniken wäre ein wichtiger Hinweis für weitere Bemühungen um eine rationale antimikrobielle Therapie.

## Methoden

Die jeweils jüngsten Quartalsdaten zum Antibiotikaeinsatz urologischer Fachabteilungen aus dem Zeitraum 2022/23 der 323 an dem ADKA-if-DGI-Projekt teilnehmenden Kliniken wurden als Jahresdaten ausgewertet. Die berechneten Verbrauchsdaten basieren auf den aus dem Materialwirtschaftssystem der Apotheken abgegebenen Mengeneinheiten. Die Abgabemengen aller systemischen Antibiotika (nach der ATC [„anatomisch-therapeutisch-chemische“]-Klassifikation auf der Ebene „J01“) wurden kostenstellengenau erfasst, nach Qualitäts- und Plausibilitätsprüfung zusammen mit den zugehörigen Belegungsdaten in eine Datenbank eingelesen und als Verbrauchsdichte (Tagesdosen pro 100 Pflegetage) auswertet.

Als Tagesdosen wurden krankenhausadaptierte, von uns in früheren Untersuchungen [16, 17] validierte Dosierungen verwendet („recommended daily doses“, RDD, Stand 20.06.2024). Sie unterscheiden sich von den seitens der Weltgesundheitsorganisation (WHO) im dortigen Index gemäß Anatomisch-Therapeutisch-Chemischem Klassifikationssystem (ATC) (https://atcddd.fhi.no/atc_ddd_methodology/who_collaborating_centre/) vermerkten definierten Tagesdosen („defined daily doses“, DDD) in erster Linie bei den Betalaktam-Antibiotika, deren Tagesdosis nach ATC-DDD-Index oft niedriger ist als bei stationären Patientinnen und Patienten empfohlen und verordnet.

Zum Analysezeitpunkt waren die Daten von 107 urologischen Fachabteilungen mit vollständigen Daten zu mindestens vier aufeinanderfolgenden Quartalen für eine deskriptive Auswertung verfügbar. Die Auswertungen wurden stratifiziert nach Krankenhausgröße bezogen auf die Bettenzahl der Gesamtklinik (nichtuniversitäre Krankenhäuser < 400 [*n* = 25], 400–800 [*n* = 39], > 800 [*n* = 22] Betten) bzw. nach Status als Universitätsklinik (*n* = 21). Unterschiede in der Verbrauchsdichte und in den relativen Anteilen bestimmter Wirkstoffe wurden mittels nicht-parametrischer Tests (nach Kruskal-Wallis) auf statistische Signifikanz geprüft.

Die Sammlung und Auswertung der epidemiologischen Daten über das Surveillance-Programm erfolgt in Übereinstimmung mit geltenden Rechtsvorschriften in Deutschland. Es wurden keine Studien an Menschen oder Tieren durchgeführt oder personenenbezogene Daten erfasst. Eine ethische Überprüfung ist daher nicht erforderlich.

## Ergebnisse

**Abb. 1:**
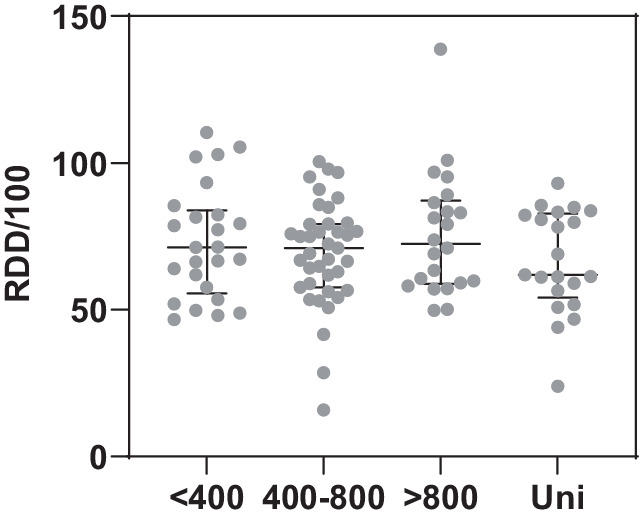
Die Verteilung der Antibiotikaverbrauchsdichte (in RDD/100 [„recommended daily doses“]) für 107 urologische Fachabteilungen (2022/23) nach Krankenhausgrößenklasse (Bettenzahl bzw. Status als Universitätsklinik). Dargestellt sind je Gruppe zusätzlich der Median und Interquartilbereich. Die Unterschiede zwischen den Gruppen waren statistisch nicht signifikant (*p* = 0,7146, Kruskall-Wallis-Test)

Die mediane AD in den 107 Fachabteilungen betrug insgesamt 71 RDD/100 mit einer Spannweite zwischen 15,9 und 138,7 und einem Interquartalbereich (IQR) von 57,9–82,7 (Tab. 1). Die Gesamtverbrauchsdichte unterschied sich wenig nach Krankenhausgröße (Abb. 1). Es zeigte sich eine Tendenz zu einer niedrigeren Verbrauchsdichte in den universitären Fachabteilungen (61,9 RDD/100 [Median] versus 71,0–72,4 in den nichtuniversitären Fachabteilungen; Abb. 1). Die Unterschiede waren jedoch statistisch nicht signifikant (*p* = 0,7146). Im Vergleich zu anderen Fachabteilungen im selben Zeitraum – z. B. Allgemeine Innere Medizin (mediane Verbrauchsdichten je nach Krankenhausgröße 39–47–RDD/100), Gynäkologie (mediane Verbrauchsdichten je nach Krankenhausgröße 17–25 RDD/100) oder Allgemeinchirurgie (mediane Verbrauchsdichten je nach Krankenhausgröße 42–61 RDD/100) – war die Verbrauchsdichte in der Urologie vergleichsweise hoch (unveröffentlichte Daten).

**Tab. 1:** Antibiotikaverbrauchsdichte (in RDD/100 [„recommended daily doses“]) in 107 urologischen Fachabteilungen insgesamt und für ausgewählte (die häufigsten 8) Wirkstoffklassen/Wirkstoffe mit ihren relativen Anteilen pro Fachabteilung an der Gesamtverbrauchsdichte (*3°/4°-Cephalosporine* Cephalosporine der dritten und vierten Generation, *1°/2°-Cephalosporine* Cephalosporine der ersten und zweiten Generation, *IQR* Interquartilbereich)

	Verbrauchsdichte (RDD/100)				
Wirkstoff(gruppe)	Median	IQR	Spannweite (min–max)	Relative Anteile	
				Median (%)	IQR (%)
Gesamt	71,0	57,9–82,7	15,9–138,7		
3°/4°-Cephalosporine	16,2	6,8–21,4	0,5–74,3	22	(12–34)
Aminopenicillin/Betalaktamaseinhibitor	10,8	7,0–21,3	0,2–69,6	16	(11–26)
Breitspektrumpenicilline	8,9	6,6–11,8	0–31,1	13	(9–17)
Fluorchinolone	6,0	3,3–9,9	0–28,6	9	(5–13)
Cotrimoxazol	4,0	1,7–6,6	0–23,8	6	(3–9)
1°/2°-Cephalosporine	3,9	1,8–11,9	0–38,9	6	(3–15)
Carbapeneme	3,4	2,2–4,7	0–9,3	5	(3–7)
Schmalspektrumpenicilline	1,3	0,7–2,3	0–12,2	2	(1–3)

*IQR* Interquartilbereich, *RDD* „recommended daily doses“

**Abb. 2:**
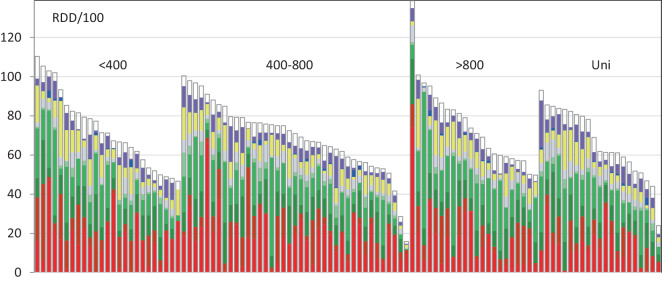
Die Verordnungsdichten in allen 107 urologischen Fachabteilungen, sortiert nach Krankenhausgrößenklasse (Bettenzahl bzw. Status als Universitätsklinik). Dargestellt sind die unterschiedlichen Wirkstoffklassen (addiert zum Gesamtverbrauch pro Fachabteilung): *hellrot* Cephalosporine der dritten/vierten Generation; *dunkelrot* Cephalosporine der ersten/zweiten Generation; *dunkelgrün* Breitspektrum-Penicilline; *grün* Aminopenicillin/Betalaktamaseinhibitor-Kombinationen; *hellgrün* Schmalspektrum-Penicilline; *gelb* Fluorchinolone; *blau* Aminoglykoside; *violett* Cotrimoxazol; *weiß* sonstige

**Abb. 3:**
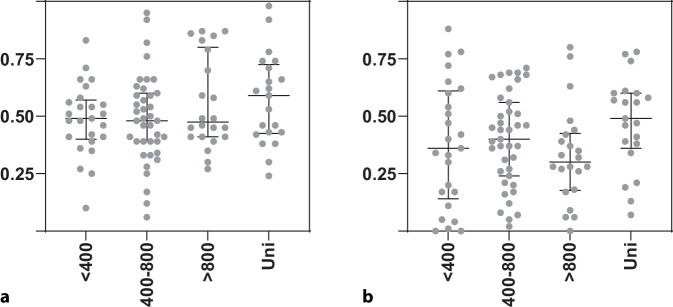
Die Relationen in der Verordnungsmenge (in RDD) von (**a**) Penicillinen zu Cephalosporinen und von (**b**) Cotrimoxazol zu Fluorchinolonen nach Krankenhausgrößenklasse (Bettenzahl bzw. Status als Universitätsklinik). Angezeigt sind je Gruppe zusätzlich der Median und Interquartilbereich. Die Unterschiede zwischen den Gruppen waren statistisch nicht signifikant (*p* = 0,4458 [**a**] bzw. *p* = 0,1358 [**b**], Kruskall-Wallis-Test)

Bei Betrachtung der verschiedenen Wirkstoffklassen zeigte sich, dass Betalaktame einen hohen Anteil der Verordnungen ausmachen (insgesamt 75 %). Unabhängig von der Krankenhausgröße waren dabei Cephalosporine der dritten und vierten Generation (darunter 64 % Ceftriaxon) und Aminopenicillin/Betalaktamaseinhibitor-Kombinationen die am häufigsten verordneten Substanzen (Tab. 1 und Abb. 2). Breitspektrumpenicilline (mit Piperacillin-Tazobactam und Piperacillin), Fluorchinolone und die anderen Wirkstoffklassen bzw. Wirkstoffe wurden seltener verordnet, und deren Spannweite war erheblich (Tab. 1). Der relative Anteil der Cephalosporine der dritten und vierten Generation am Gesamtverbrauch der Fachabteilungen betrug im Median > 20 % (Tab. 1). Insgesamt wurden Penicillin-Derivate (am häufigsten i.v. Ampicillin-Sulbactam oder p.o. Amoxicillin-Clavulansäure) jedoch häufiger eingesetzt als Cephalosporine. Über alle Fachabteilungen hinweg (als gewichteter Mittelwert) war das Verhältnis zwischen Penicillinen zu Cephalosporinen (in Tagesdosen) 52:48. Die Schwankungen waren allerdings extrem (zwischen 6:94 und 98:2). Statistisch signfikante Unterschiede im Cephalosporin:Penicillin-Verhältnis zwischen den Fachabteilungen der unterschiedlichen Krankenhausgrößen waren nicht darstellbar (Abb. 3).

Fluorchinolone (darunter 70 % Ciprofloxacin) waren die viertstärkste Substanzklasse (mediane Verordnungsdichte 6,0 RDD/100, Spannweite 0–28,6, IQR 3,3–9,9). Ihr Anteil an der Gesamtverordnungsmenge pro Fachabteilung lag < 10 % im Median (Tab. 1). Die Relation Cotrimoxazol zu Fluorchinolonen pro Fachabteilung schwankte stark (Abb. 3). Sie betrug (im gewichteten Mittel) über alle Fachabteilungen zusammen 40:60. Aminoglykoside wurden extrem selten verordnet (mediane Verordnungsdichte 0,1 RDD/100, Anteil am Gesamtverordnungsvolumen < 1 %). Die fünf verordnungsstärksten Wirkstoffe unter den parenteralen Antibiotika (nach abnehmender RDD-Menge) waren Ceftriaxon, Piperacillin-Tazobactam, Ampicillin-Sulbactam, Cefuroxim und Meropenem. Cefotaxim (als Alternative zu Ceftriaxon) wurde wenig verordnet. Auch die in der Urologie relevanten parenteralen, so genannten Reserveantibiotika Ceftazidim-Avibactam und Cefiderocol wurden sehr selten verordnet, ebenso parenterales Fosfomycin (Anteile an allen verordneten Tagesdosen jeweils < 0,1 %).

Der Anteil oraler Antibiotika am Gesamtverbrauch war in den Krankenhausgrößenklassen ähnlich (Mediane zwischen 40,5 und 47,6 %) und lag für alle Fachabteilungen zusammen (als gewichteter Mittelwert) bei 44,7 % (Tab. 2). Er zeigte in einer einfachen linearen Regression einen nur geringen Zusammenhang mit der Gesamtverbrauchsdichte (R^2^ = 0,0497). Die verordnungsstärksten oralen Antibiotika waren (nach abnehmender RDD-Menge) Amoxicillin-Clavulansäure, Cotrimoxazol, Cefpodoximproxetil, Ciprofloxacin und Cefuroximaxetil, gefolgt von Levofloxacin und Amoxicillin (Tab. 3). Alle anderen oral applizierten Wirkstoffe, insbesondere auch Fosfomycin, Pivmecillinam, Nitrofurantoin, Nitroxolin, Cefixim sowie sonstige (neben Cefpodoximproxetil und Cefuroximaxetil) Oralcephalosporine (Cefaclor, Cefalexin, Cefadroxil) und Norfloxacin wurden sehr viel seltener verordnet (jeweiliger Anteil am Gesamtverbrauch < 1 %). Andere Antibiotika, wie z. B. Metronidazol, Tetrazykline, Makrolide, Linezolid u. a., wurden ebenfalls sehr selten verordnet.

**Tab. 2:** Verordnungsdichte oraler Antibiotika (in RDD/100 [„recommended daily doses“]) in 107 urologischen Fachabteilungen nach Krankenhausgrößenklasse (Bettenzahl bzw. Universitätsklinik) insgesamt sowie für ausgewählte Wirkstoffklassen/Wirkstoffe mit ihren relativen Anteilen am Gesamtverbrauch (parenteral plus oral) als gewichtete Mittelwerte für alle Kliniken zusammen (*3°/4°-Cephalosporine* Cephalosporine der dritten und vierten Generation, *1°/2°-Cephalosporine* Cephalosporine der ersten und zweiten Generation, *Uni* Universitätsklinik, *p.o.* per os

p.o. Wirkstoff(gruppe)	Verbrauchsdichte (RDD/100) nach Bettenzahl bzw. Universitätsklinik (Mediane) und in allen Fachabteilungen						Anteile (%) am Gesamtverbrauch
	< 400	400–800	> 800	Uni	Gesamt		
					Median	IQR	
Gesamt	32,7	31,5	27,8	29,7	29,5	20,9–40,7	44,7
3°/4°-Cephalosporine*	6,6	3,7	1,2	1,7	3,0	0,3–7,8	6,5
1°/2°-Cephalosporine*	1,0	0,6	2,4	0,2	0,8	0,0–3,5	3,6
Aminopenicillin/Betalaktamaseinhibitor*	6,9	6,0	7,1	8,2	6,5	3,8–12,1	13,6
Schmalspektrumpenicilline*	0,9	1,2	0,5	0,8	0,9	0,5–1,8	2,1
Fluorchinolone*	4,5	5,4	5,7	4,1	5,0	2,8–8,3	8,3
Cotrimoxazol	3,7	3,2	2,5	4,0	3,5	1,4–5,9	6,2

*Die jeweilig verordnungsstärksten Substanzen innerhalb der verschiedenen oral verabreichten Wirkstoffklassen waren: Cefpodoximproxetil, Cefuroximaxetil, Amoxicillin-Clavulansäure, Amoxicillin, Ciprofloxacin

*IQR* Interquartilbereich, *RDD* „recommended daily doses“

**Tab. 3:** Anteil der häufigsten^a^ oralen Antibiotika (in % des Gesamtverbrauchs) in 107 urologischen Fachabteilungen als gewichtete Mittelwerte für alle Kliniken zusammen (*p.o.* per os)

p.o. Wirkstoff	Anteile (%) am Gesamtverbrauch
Amoxicillin/Clavulansäure	12,7
Cotrimoxazol	6,2
Cefpodoximproxetil	6,2
Ciprofloxacin	6,2
Cefuroximaxetil	3,4
Levofloxacin	2,0
Amoxicillin	1,5

^a^Wirkstoffe mit einem relativen Anteil von > 1 %

## Diskussion

Die vorliegende Querschnittanalyse des Antibiotikaverbrauchs in urologischen Fachabteilungen deutscher Krankenhäuser zeigt eine relativ hohe Anwendungsdichte. Die Schwankungen zwischen den Abteilungen sind – mit einer Spannweite zwischen < 20 und > 100 RDD/100 – sehr groß und nicht nachweisbar beeinflusst von der Krankenhausgröße bzw. dem Status als Universitätsklinik. Strukturelle Besonderheiten wie spezielle Operations- bzw. Behandlungsschwerpunkte, größere Unterschiede im Patientenmix bezüglich Alter und Begleitkrankheiten sowie Abteilungsgröße und -einbindung etc. können diese Variabilität teilweise erklären. Wir betrachten als eine der wichtigsten Botschaften dieser Analyse, dass extrem hohe Verbrauchsdichten – wie hier beobachtet, z. B. valide außerhalb des Interquartilbereichs – Anlass geben sollten, die Verschreibungsgewohnheiten vor Ort und eventuelle Besonderheiten sorgfältig zu erfassen und zu beurteilen und ggf. Maßnahmen zu einer optimierten und rationalen Verordnung einzuleiten. Erfahrungsgemäß sind es vielfach verlängerte Therapien oder auch verlängerte perioperative Antibiotikaprophylaxen, die eine ungewöhnlich hohe Verbrauchsdichte verursachen [18–23].

Ein wichtiger Befund der vorliegenden Auswertung ist die aktuell große Bedeutung der Betalaktam-Antibiotika und die demgegenüber sehr viel geringere Bedeutung der Fluorchinolone in der stationären Patientenbetreuung in der Urologie. Längsschnittstudien zeigen, dass in vielen medizinischen Bereichen die Fluorchinolon-Verordnungen zurückgegangen sind [4] – primär in Folge der wiederholten Warnhinweise aufgrund teilweise schwerer unerwünschter Arzneimittelwirkungen [24]. Fluorchinolone machen nach unseren Daten meist nicht mehr als 10 % aller Antibiotikaverordnungen in der stationären Urologie aus, und ihre Verordnungsdichte pro Fachabteilung ist (bei einem Median von 6 RDD/100) selten > 10 RDD/100. Oft werden sie in der oralen Form verordnet. Hier haben sie jedoch keine Sonderstellung mehr. Zahlreiche alternative Wirkstoffe stehen zur Verfügung und werden offenbar auch eingesetzt, namentlich Amoxicillin-Clavulansäure und – seltener – Cotrimoxazol oder Cefpodoximproxetil. Diese alternativen Substanzen sind grundsätzlich zur oralen Therapie von verschiedenen Formen der Harnwegsinfektion geeignet, wenn auch nicht immer als Therapie der Wahl empfohlen und eher im Sinne der gezielten Therapie (nach Erregersicherung und Austestung) vorgesehen. Es war überraschend zu sehen, dass Amoxicillin-Clavulansäure häufiger eingesetzt wurde als Cotrimoxazol – angesichts der günstigeren Resistenzsituation von *Escherichia coli* gegenüber Cotrimoxazol (15–35 % vs. 30–40 %) war dies nicht erwartet. Möglicherweise spielen Lieferengpässe hier eine Rolle. Möglicherweise spielen auch Enterokokken als Erreger nosokomialer Infektionen eine Rolle, da diese als resistent gegenüber Cotrimoxazol gelten. Auffällig war bei einem Anteil von immerhin rund 5 % Carbapeneme an allen Verordnungen der sehr geringe Anteil von (sonstigen) Reserveantibiotika wie Ceftazidim-Avibactam und Cefiderocol, aber auch von Aminoglykosiden und parenteralem Fosfomycin.

Der Anteil der oralen Antibiotika ist mit rund 45 % in der stationären Urologie relativ hoch. Die damit verbundenen Fragen der Notwendigkeit einer (weiteren) stationären Behandlung (vs. Entlassung) sind nicht unerheblich. Gründe für eine weiter bestehende stationäre Behandlungspflicht trotz bereits erfolgter Oralisierung einer Antibiotikatherapie können vielfältig sein, und wir halten es für richtig, dass eine orale Therapieoption fachlich begründet bleibt und weitgehend unabhängig von Kriterien der Notwendigkeit einer Krankenhausbehandlung indiziert wird. Verkürzte Verweildauern aufgrund einer frühen Oralisierung mit Entlassung führen in der Regel auch zu einer höheren Antibiotika-Verbrauchsdichte, da die Fallzahlen und damit die Behandlungsintensität steigen. Sehr hohe Verbrauchsdichten in unserer Erhebung in einzelnen Abteilungen könnten dadurch begründet sein. Gemäß den Daten einer früheren internationalen Prävalenzstudie war der Anteil der oralen Antibiotika in der stationären Urologie in Deutschland vergleichsweise hoch [25]. Aktuelle Daten mit größeren Fallzahlen hierzu und Analysen zu den Effekten auf kürzere Verweildauern hierzulande fehlen jedoch. Eine jüngere monozentrische Erhebung aus Deutschland zeigte einen mutmaßlichen Anteil oraler Antibiotika in der Urologie von rund 50 % [21]. Eine ältere ebenfalls monozentrische Studie aus den Niederlanden zeigte eine Oralisierungsquote von < 50 % [26].

Aus Gründen der Toxizität und/oder der Kollateralschäden im Sinne einer begünstigten Resistenzentwicklung und höherer Superinfektionsrisiken (z. B. durch *Clostridioides difficile*) werden Fluorchinolone aber auch Cephalosporine, v. a. solche mit hoher biliärer Ausscheidung oder schlechter Resorption, inzwischen sehr zurückhaltend empfohlen. Es gibt geeignete und vergleichbar effektive Therapiealternativen [7, 14, 15, 27]. In diesem Zusammenhang kann es von Interesse sein, Ziele für den relativen Einsatz solcher Substanzen im Vergleich zu den Alternativen zu formulieren. Das Verhältnis Penicilline zu Cephalosporinen und Cotrimoxazol zu Fluorchinolonen, wie wir es analysieren und hier vorstellen konnten, könnte eine Hilfe sein. So war der Median beim Penicillin-Cephalosporin-Verhältnis in dieser Studie 52:48 (oder 52 %). Fachabteilungen mit einer geringeren Rate könnten Gründe dafür evaluieren und ggf. Therapieempfehlungen anpassen, implementieren und mögliche Interventionseffekte mit einem solchen relativ einfachen Indikator im Verlauf beurteilen. Bemerkt werden soll, dass die Relation Penicilline zu Cephalosporinen in deutschen Krankenhäusern (disziplin-/abteilungsübergreifend) in den letzten Jahren stark angestiegen ist und inzwischen bei > 70 % liegt (unveröffentlichte Daten). Ein ähnlicher Indikator, zu dem bisher weniger Erfahrung existiert, könnte das Verhältnis zwischen Cotrimoxazol und Fluorchinolonen werden. Aber auch die Auswahl des Cephalosporins ist von Bedeutung. Ceftriaxon – der am häufigsten verwendete Wirkstoff in unserer Erhebung – ist aufgrund der obigen Überlegungen gegenüber Cefotaxim vermutlich nachteilig – auch wenn es hierfür keine aussagekräftigen direkten Vergleichsstudien gibt.

Zwei weitere Beobachtungen dieser Studie verdienen eine kurze Bemerkung. Die Studie belegt, dass Antibiotika mit der Indikation (unkomplizierte) Zystitis in der stationären Urologie primär keine große Rolle spielen und Therapieempfehlungen hierzu v. a. auch an Notfallambulanzen, Aufnahmestationen und Rettungsstellen (neben der Allgemeinmedizin) adressiert werden müssen. Ein zweiter Befund von Interesse könnte die sehr niedrige Verordnungshäufigkeit von Aminoglykosiden sein. Aminoglykoside wie Gentamicin und Tobramycin sind hierzulande bei zahlreichen Uropathogenen noch sehr gut aktiv und können durchaus als eine Therapiealternative in Betracht gezogen werden. Zwar ist die Toxizität zu berücksichtigen, aber das Risiko hierfür ist bei Kurzzeittherapien gering. Erfahrungen aus anderen Ländern mit ausgewählten Patienten zu einer initialen empirischen kurzen Behandlung einer Harnwegsinfektion bis weitere Befunde vorliegen, sind ermutigend – auch in der Monotherapie [28–30]. Ähnlich verhält es sich mit parenteralem Fosfomycin, das ein geringes Toxizitätsrisiko hat.

Stärken dieser Untersuchung ist die große Anzahl von teilnehmenden Krankenhäusern und die sorgfältige Prüfung der Daten auf Plausibilität und Vollständigkeit. Dennoch können Dokumentations- und Buchungsfehler nicht immer entdeckt werden. Eine Limitation dieser Analyse ist der fehlende Patientenbezug. Inwieweit die Substanzen für empirische oder gezielte Therapie oder für die Prophylaxe verwendet wurden, und inwiefern Unterschiede in der Anwendungshäufigkeit durch unterschiedliche Diagnosespektren und auch unterschiedliche lokale Resistenzsituationen mitbedingt sind, kann damit nicht beurteilt werden. Wertvoll für die weitere Bewertung insgesamt und auch vor Ort in den Fachabteilungen wären Beobachtungen im Längsschnitt. Einen residualen Einfluss der Pandemie auf die Krankenhausstruktur, Belegungsmuster und Arzneimittelverordnungspraxis können wir ebenfalls nicht ganz ausschließen.

Zusammenfassend lässt sich aktuell eine variable, insgesamt jedoch vergleichsweise hohe AD in stationären urologischen Fachabteilungen in Deutschland beobachten. Fluorchinolone werden im Mittel wenig eingesetzt. Es dominieren Betalaktame, oft Ceftriaxon, überraschend häufig aber auch Amoxicillin-Clavulansäure in der oralen Applikationsform. Fachabteilungen mit relativ hoher Verbrauchsdichte (beispielsweise oberhalb der 75 %-Perzentile) und deutlicher Bevorzugung von Cephalosporinen (gegenüber Penicillinen) und/oder von Fluorchinolonen (gegenüber Cotrimoxazol) können und sollten die Daten für Benchmarking-Zwecke verwenden und ggf. ihre Therapieempfehlungen anpassen und Leitlinienadhärenz-fördernde Maßnahmen intensivieren. Eine Diskussion der Befunde zum Antibiotikaverbrauch und zu den lokal gemessenen Resistenzraten der wichtigsten Erreger sollte regelmäßig erfolgen und auch die älteren Wirkstoffe wie Aminoglykoside und parenterales Fosfomycin als mögliche Behandlungsoptionen bei ansonsten resistenten Uropathogenen einbeziehen.

## Fazit für die Praxis

- Die Antibiotikaverbrauchsdichte in den 107 Fachabteilungen betrug im Median 71 Tagesdosen pro 100 Pflegetage und schwankte erheblich (zwischen < 20 und > 100).
- Betalaktame waren die am häufigsten verordneten Wirkstoffe; Breitspektrum-Cephalosporine (mit Ceftriaxon und Cefpodoximproxetil als häufigste Substanzen) wurden etwas häufiger verordnet als Aminopenicillin/Betalaktamaseinhibitor-Kombinationen. Das Verhältnis zwischen Penicillinen und Cephalosporinen schwankte zwischen 6:94 und 98:2 (insgesamt 52:48).
- Cotrimoxazol wurde etwas seltener verordnet als Fluorchinolone. Beide Substanzen wurden überwiegend oral verabreicht. Der Anteil oraler Antibiotika betrug insgesamt 44,7 % und schwankte nach Krankenhausgröße wenig. Dabei wurden Fosfomycin, Pivmecillinam, Nitrofurantoin und Nitroxolin deutlich seltener verordnet als orale Betalaktame, Fluorchinolone und Cotrimoxazol.
- Die sehr selten verordneten Aminoglykoside und parenterales Fosfomycin könnten als mögliche Behandlungsoptionen bei ansonsten resistenten Uropathogenen zur Einsparung von klassischen Reserveantibiotika berücksichtigt werden.
- Fachabteilungen mit relativ hoher Verbrauchsdichte und deutlicher Bevorzugung von Cephalosporinen und/oder von Fluorchinolonen sollten die Daten für Benchmarking-Zwecke verwenden und ggf. ihre Therapieempfehlungen reevaluieren.

## Data Availability

Die erhobenen Datensätze können auf begründete Anfrage in anonymisierter Form beim korrespondierenden Autor angefordert werden. Die Daten befinden sich auf einem Datenspeicher am Universitätsklinikum Freiburg.
